# Experimental Evaluation and Prediction of the Dynamic Modulus of Crumb Rubber-Modified Stone Mastic Asphalt Mixtures

**DOI:** 10.3390/polym18101249

**Published:** 2026-05-20

**Authors:** Muhammad Irfan, Saif Ullah Khan Wazir, Muhammad Asif Khan, Sarfraz Ahmed, Zain Maqsood

**Affiliations:** 1 School of Civil and Environmental Engineering (SCEE), National University of Sciences & Technology (NUST), Islamabad 44000, Pakistan; muhammad.asif@nice.nust.edu.pk (M.A.K.); zmaqsood@nice.nust.edu.pk (Z.M.); 2 Military College of Engineering (MCE), National University of Sciences & Technology (NUST), NUST Campus, Risalpur 24080, Pakistan; skwazir10@gmail.com (S.U.K.W.); sarfraz@mce.nust.edu.pk (S.A.)

**Keywords:** stone mastic asphalt, crumb rubber, dynamic modulus, master curves, performance modeling, artificial neural networks

## Abstract

Increased and excessive axle loads (exceeding design specifications) at high temperatures stimulate premature distresses in flexible pavements. This study utilizes the novelty of engineered bituminous composite—crumb rubber-modified (CRM) stone mastic asphalt (SMA) for pavement longevity and sustainable performance. Dynamic modulus testing was employed at four temperatures and six frequency sweeps. The experimental design included the preparation of SMA 19 specimens with six different percentages of crumb rubber (CR) mixed in bitumen. CR addition to the mix translated into an improved stiffness of the mix, as a 64% increase in dynamic modulus (on average) was reported at 10% CR as compared to a neat mixture. Master curves were produced using |E*| test results, which revealed that 10% modified SMA was relatively stiffer and more rut-resistant than the other mixtures. Performance prediction models were developed for |E*| using artificial neural networks (ANNs) and non-linear regression, wherein the former proved to be more robust. Sensitivity analysis revealed that a temperature rise (21.1 to 37.8 °C) translated into a 65% drop in |E*| (on average) and a rise in frequency (0.1 to 25 Hz) divulged a 72% upsurge in |E*| (on average). This research demonstrates the promise of deploying CR SMA mixtures, particularly for high-traffic and heavy-load scenarios.

## 1. Introduction

Stone mastic asphalt (SMA) is characterized by its stone-on-stone contact that is produced by the coarse aggregate structure, which helps sustain load and reduce rutting [1,2]. SMA has gained recognition all over the world for use in heavily trafficked areas [2]. Stabilizing additives and recycled textile fibers are used to reduce the binder drain and improve the resistance against the rutting and cracking of SMA mixtures [3,4,5]. The performance of SMA is better than that of HMA, and the higher values of dynamic modulus are reported for SMA mixtures as compared to the HMA mixtures [6]. SMA has low noise with satisfactory skid resistance [7].

Engineered bituminous composites play a critical role in pavement engineering by improving the material properties to meet increased traffic loading demands and climatic variability. Bitumen governs the aging behavior and viscoelastic response of asphalt concrete mixtures, despite being used in small proportions [8]. Its inherent temperature- and time-dependent behavior makes engineering interventions essential to mitigate cracking at low temperatures and rutting at high temperatures [9]. Consequently, engineered modifications to bitumen incorporating recycled materials, crumb rubber (CR) additives, and polymers have been widely implemented to enhance the durability and elasticity of asphalt pavements across a wide range of temperatures [10,11,12]. Such engineered composites are considered effective solutions for improving pavement performance, extending service life, and addressing sustainability challenges related to material costs and environmental impacts [13,14,15].

CR is an elastomer produced from waste tires that have completed their service life, so its usage in SMA warrants a reduction in the dumping of this waste, which pollutes the environment [16]. The use of CR as a modifier helps address the Sustainable Development Goals by using waste material. Research shows that CR increases the stiffness, temperature stability, and pavement service life, reduces the desired thickness of pavement, improves the behavior of asphalt against rutting and reflective cracking, reduces noise pollution, and improves the skid resistance of the pavement [7,16,17,18,19].

Kök et al. [20] studied CR-modified (CRM) SMA mixtures and tested them for stiffness, permanent deformation, fatigue, and moisture susceptibility. CRM SMA mixtures performed better compared to control mixtures containing an unmodified binder. Rutting and fatigue resistance, along with moisture susceptibility, was investigated for CRM SMA by Xie and Shen. Improved rutting resistance was observed at 10% crumb rubber content under the specific test conditions [21]. Mashaan et al. [22] studied the stiffness and fatigue resistance of CRM SMA mixtures to reduce construction costs associated with expensive polymers. The modified SMA samples showed a higher stiffness modulus (3996 MPa) than neat mixtures (1370 MPa) without rubber at 5 °C. The fatigue life of modified SMA samples was significantly higher than that of non-modified samples. Tai Nguyen and Nhan Tran [23] investigated the effect of curing time and CR content on the mechanical properties, especially the rutting performance of CRM asphalt concrete and SMA mixtures. The results revealed that adding 1.5 to 2% CR by weight of the mixture and keeping the curing time at 0 to 5 h (the longer the better at high temperatures) results in optimal performance of both mixtures regarding rutting resistance and other mechanical properties. CR was incorporated into SMA to test its potential for use in the heavy traffic and hot temperatures (20 °C to 30 °C) of Angola. It was also investigated whether adding rubber to the mix would waive the requirement for cellulose fibers, as they are not locally available. The mixture’s susceptibility to moisture was tested, and its resistance to fatigue and pavement deformation was evaluated. It was concluded that rubberized SMA performed well at high temperatures and provided effective, durable pavements without the need to import any additional material [24]. Noura et al. [25] utilized truck tire rubber as a modifier in SMA using wet and dry processes. The mixtures modified by the wet process exhibited higher elasticity, as their phase angle values were lower than those of SMA mixtures prepared with the dry process. The addition of CR to SMA results in improved fatigue life, better aging resistance, and reduced temperature susceptibility. Han et al. measured the short-term stress relaxation behavior of the CRM asphalt binder under a wet process within a linear viscoelastic region. The study results indicated that the maximum critical strain of CRM asphalt was approximately 1% at 10–15 °C [26]. Gardezi and Hussain used scrap tire rubber in SMA and replaced cellulose fibers with munjin fiber to test the performance of SMA-25. Resistance to moisture susceptibility improved with the addition of CR to SMA [27]. Ameli et al. [28] tested the performance of CRM SMA using resilient modulus, dynamic creep, four-point beam fatigue, ITS, and the wheel tracker test. The tests concluded that CR enhanced the properties of the SMA mixture with an increased resilient modulus and ITS. The resistance offered to fatigue and rutting also improved with the inclusion of CR. A study by Jebur et al. [29] evaluated the performance of SMA modified with CR (5% to 20%) as a binder modifier and cellulose fiber pellets (CFP) as a stabilizing agent. The drain down test, resilient modulus, tensile strength ratio, and fatigue life were used as performance indicators. The authors concluded that adding 15% CR and 0.4% CFP yields the optimal performance of the modified SMA samples across all performance indicators. Zakerzadeh et al. [30] recently conducted a detailed review study investigating the effect of using waste tire rubber in SMA. The authors identified and discussed research gaps related to aggregate gradation, processing temperature, binder content, compaction technique, and the CR grain-size distribution and its concentration. The authors also highlighted that key performance indicators considered in the CRM SMA literature include moisture susceptibility, binder drain down, fatigue cracking, and permanent deformation (rutting), with dynamic modulus as a performance indicator missing. Calabi-Floody et al. [31] validate the feasibility of a waste tire textile fiber (WTTF) additive as a sustainable substitute for cellulose in SMA mixtures. The WTTF-modified SMA demonstrated superior mechanical performance, including higher fatigue durability and improved moisture resistance.

Some recent studies have used machine learning models to predict the dynamic modulus of asphalt concrete mixtures. For instance, Ali et al. [32] used an eXtreme Gradient Boosting (XGBoost) machine learning model to predict the dynamic modulus of HMA samples. The authors considered testing conditions, the mix’s volumetric properties, and gradation type as input parameters to the model. The results were also compared with the other well-known regression and machine learning (ML) models used to predict the dynamic modulus. The results indicated that the XGBoost model outperforms the other models. The authors suggested using deep neural networks to more accurately predict the dynamic modulus. Moussa and Owais [33] conducted a study to predict the dynamic modulus of HMA using deep residual neural networks (DRNNs). The model outputs were compared with those of other commonly employed dynamic modulus prediction models: the Hirsch and Witczak (1-37A and 1-40D) models. The results revealed that DRNNs outperformed the other models, and the testing temperature and the binder’s stiffness characteristics were the most significant factors influencing the dynamic modulus. Hussain et al. [34] developed an artificial neural network (ANN) model to predict the behavior of differential phase angle for wearing versus base course mixes using laboratory test data on 23 AC mixtures. The AC mixtures, comprising binders with different penetration grades, mix types, and mix proportions, were used to test the phase angle at different temperatures and loading frequencies. The results of the developed ANN model were also compared with other linear and non-linear models, and the ANN outperformed in accurately predicting the phase angle.

Existing studies typically examine the effect of fixed CR percentages without assessing the influence of varying modifier content. For instance, Morea et al. [35] evaluated the effects of a fixed percentage of CR (22%) for the investigation of the dynamic modulus of high-viscosity CRM SMA without fibers, through the ITS method. Similarly, Xie and Shen [21] investigated the dynamic modulus of CRM SMA with a 10% modifier content and an SMA 12.5 gradation, considering only three temperature levels (4, 20, and 45 °C) and four frequency bands (0.01, 0.1, 1.0, and 10 Hz). The present study fills this research gap by focusing on SMA 19 gradation mixtures and evaluating their dynamic modulus behavior under an expanded combination of temperatures (4.4 °C, 21.1 °C, 37.8 °C, and 54.4 °C) and loading frequencies (0.1, 0.5, 1, 5, 10, and 25 Hz) using the Asphalt Mixture Performance Tester (AMPT). Thus, it better simulates field conditions and makes a significant contribution to the existing literature.

Four major conclusions can be drawn from the existing literature. Firstly, several studies have focused on evaluating the rutting resistance and fatigue performance of HMA and SMA mixtures modified with CR. Adding CR in specific proportions (5% to 20%) to both sample types enhanced fatigue life and resistance to deformation, making them more resilient across various temperatures, frequencies, and loading conditions. Secondly, some studies employed statistical and machine learning methods to predict the dynamic modulus of conventional HMA (without adding CR). Thirdly, based on the literature reviewed, limited research has evaluated and predicted the dynamic modulus (|E|) of CRM SMA mixtures using conventional statistical or ML models. Fourthly, existing studies have largely limited their investigations to fixed CR contents, narrower temperature ranges, and coarse gradation types such as SMA 12.5. The lack of research involving a broader range of CR percentages, more comprehensive temperature and frequency conditions, and finer gradation types, such as SMA 19, limits the applicability of earlier findings. This study overcomes these limitations by evaluating multiple CR contents (0–12%), using SMA 19 gradation, and simulating realistic field conditions through an extended range of testing temperatures and loading frequencies. More specifically, the study investigates the effect of varying CR content on the dynamic modulus |E*| of CRM SMA to determine the optimal CR modification percentage. The study also employed an ANN model to predict the dynamic modulus |E*| of CRM SMA, a task not previously undertaken.

## 2. Materials and Methods

**Figure 1:**
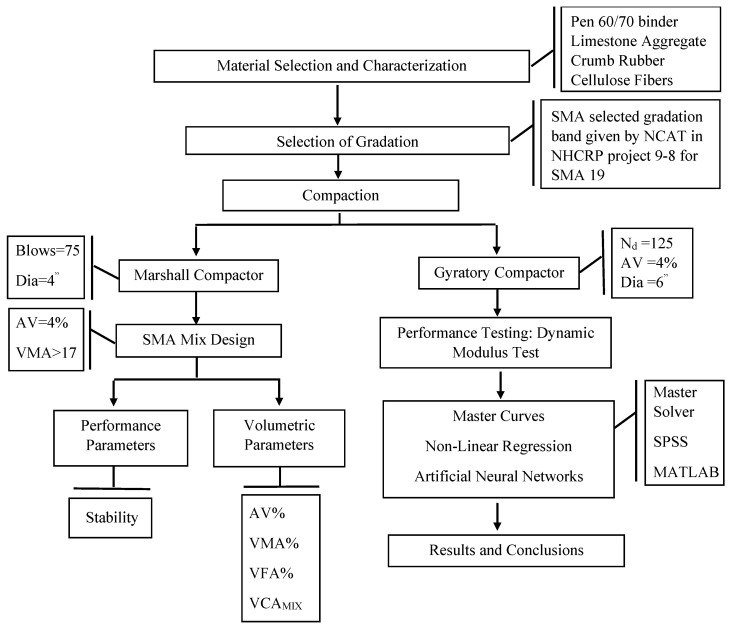
Research methodology flow chart.

The methodology adopted for the research, including material selection and characterization, is shown in Figure 1. In the first step, prerequisite data for the dynamic modulus testing of CRM SMA using an Asphalt Mixture Performance Tester (AMPT) were required. These data comprise the selection and characterization of material, including the type of binder and aggregates, gradation type, binder modifier, stabilizing additives, optimum asphalt content determination using the Marshall mix design method, and preparing modified SMA Superpave Gyratory samples using the obtained optimum asphalt content for the dynamic modulus testing. Specifically, the study employed a 60/70 penetration-grade binder, limestone aggregate, CR as a modifier, cellulose fibers as stabilizing additives, and SMA 19 gradation as specified by the National Center for Asphalt Technology (NCAT) in National Cooperative Highway Research Program (NCHRP) project 9-8. In the next step, non-linear regression and ANN models were developed to predict the dynamic modulus of CRM SMA mixtures, using temperature, frequency, and CR percentage as independent variables.

Marshall compacted specimens were fabricated to determine the gradation, bitumen content, and volumetrics of the specimens in line with the SMA mixture design as per NCHRP Report 425 [36]. Gyratory samples were fabricated using a gyratory compactor to evaluate the mixture’s performance. Triplicate specimens were prepared for the dynamic modulus test at each percentage of CRM SMA using an AMPT, and master curves were generated using the master solver Excel sheet. The results were used for the analysis and development of prediction models using ANNs.

### 2.1. Material Selection and Characterization

Materials utilized in this research include aggregates, binder, crumb rubber, and stabilizing additives/cellulose fibers.

#### 2.1.1. Aggregates

**Figure 2:**
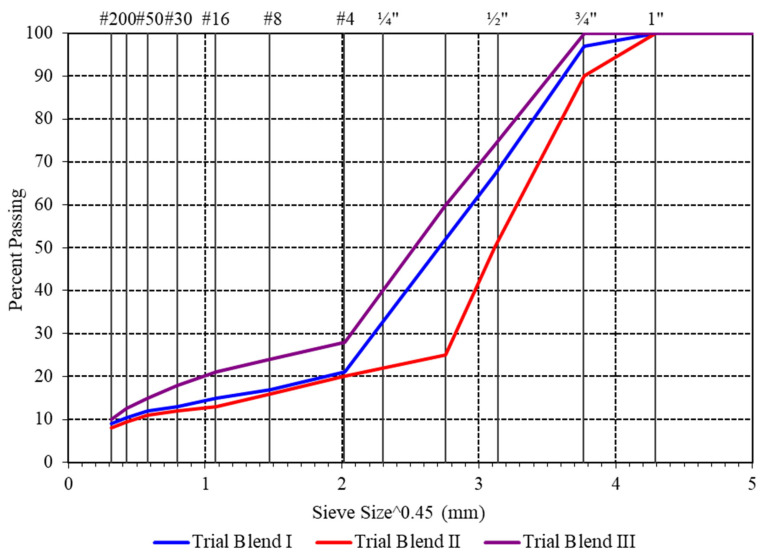
Gradation charts for SMA 19 selected gradations.

Crushed limestone aggregate was procured from the Babozai quarry site, Babozai, Pakistan and brought to the lab for testing. Aggregate characteristics hold immense importance due to the requirement of stone-on-stone contact when designing a mixture for SMA [37]. The shape and hardness of aggregates are even more crucial for SMA as compared to conventional mixtures. The properties of aggregates utilized in this research are shown in Table 1. In this study, the SMA 19 gradation band with a nominal maximum aggregate size (NMAS) of 19 mm was utilized. Three trial gradations were selected as per the criteria set in the standards and shown in the gradation chart (Figure 2) as Trial Blend I, Trial Blend II, and Trial Blend III [36]. The three trial gradations represent one at the coarse limit, one at the fine limit, and a third at the middle of the gradation band. These master specification limits for SMA gradation are given in the NCAT, NCHRP Project 9-8 [36].

#### 2.1.2. Bitumen

Bitumen was procured for this study from Attock Refinery Limited, Attock, Pakistan, with a penetration grade of 60/70. Conventional tests conducted on the bitumen are shown in Table 2 along with the results. These tests were conducted for neat and modified bitumen.

#### 2.1.3. Crumb Rubber

A local private firm, Sheikh Enterprises^®^, Lahore, Pakistan, was engaged to produce CR from end-of-life tires via an ambient grinding process, yielding particles up to 0.5 mm. CR was mixed with bitumen using the wet process for modification of the binder. It should be noted that the present study adopted the wet process for crumb rubber modification, which promotes interaction between rubber particles and bitumen at elevated temperatures; however, previous studies have reported that the dry-process incorporation of crumb rubber may result in different rheological behavior and dynamic modulus responses, which warrants further investigation [46,47]. The properties of CR used in this study are displayed in Table 3.

#### 2.1.4. Stabilizer

Stabilizing additives are added to SMA to restrict the drain down of mastic in the storage, transport, and laying of the mixture. Cellulose fibers are the most effective binder-absorbing stabilizers or drainage inhibitors to date. For this study, granulated cellulose fibers (VIATOP^®^ Premium) in the form of pellets are used, produced by J. Rettenmaier and Sohne (JRS), Rosenberg, Germany. VIATOP^®^ Premium is a pelletized blend of ARBOCEL ZZ 8/1 and Bitumen 50/70 (Table 4). It has excellent efficiency and a stabilizing effect as it provides a compact three-dimensional fiber mesh at a dosage rate of 0.3%, as listed in the specifications.

### 2.2. Preparation of Modified Binder

Crumb rubber was mixed with bitumen at 180 °C at a rotational speed of 1200 rpm for 45 min [35,48]. The temperature of the mix, mixing speed, and mixing time are critical to the proper mixing of CR into bitumen and to the formation of the CRM binder. Five different quantities of CR were added to the binder in separate batches. The percentages of CR added were 4%, 6%, 8%, 10%, and 12% (by the weight of the binder).

### 2.3. SMA Mix Design

Volumetric properties of the mix govern the design requirements of the SMA mix, which include air voids (V_a_), voids in mineral aggregate (VMA), and voids in coarse aggregate (VCA), which represent the stone-on-stone contact [49]. Three trial gradations (Figure 2) were selected from the gradation bands specified for SMA 19 [36]. VCA in the dry rodded condition (VCA_DRC_) was determined for all of the three trial gradations, Trial Blend I, Trial Blend II, and Trial Blend III, using the AASHTO T19 standard [50]. The density and VCA_DRC_ for all three trial blends selected for SMA 19 are presented in Table 5.

Specimens were prepared using a Marshall compaction machine with 75 blows per face as per the standard procedure for heavy traffic volumes. Three specimens were prepared and compacted for each trial gradation blend at 6.5% asphalt content and 0.3% fiber content. Volumetrics for all the specimens were determined for further analysis (Table 6).

Although the target air void content for SMA mixtures is 4%, Trial Blend I with 3.6% air voids was selected because it was the closest to the target value compared to Trial Blend II (3.3%) and Trial Blend III (2.9%), which deviated further from the specified requirement. It also met all SMA specification requirements (VMA > 17% and VCA < VCA_DRC_). After selecting the optimum gradation for the mix, three asphalt contents (5.5, 6.0, and 6.5%) were used, and triplicate specimens were prepared using a Marshall compaction machine with a 0.3% fiber content. The volumetrics of these specimens will govern the optimum asphalt content for the mix (Table 7).

The optimum asphalt content of 6.2% was determined by plotting Marshall volumetric properties and interpolating the asphalt content corresponding to 4% air voids, as recommended by NCHRP Report 425 for SMA mix design [36]. Additionally, all volumetric parameters and stability requirements were verified at this interpolated content, confirming compliance with established design specifications. This asphalt content is also in correspondence with the minimum asphalt content required for the mix with respect to the aggregate’s bulk-specific gravity [49]. It also satisfies all specifications for the SMA mix design outlined in NCHRP Report 425 [36]. Table 8 lists the design asphalt content and volumetrics for the final mixture.

### 2.4. Specimen Fabrication for Performance Testing

CRM SMA was evaluated using dynamic modulus as the performance indicator. Specimens were fabricated using a Superpave Gyratory compactor at a design gyration level of 125, since the mixture was designed for heavy traffic with a design ESAL ≥ 30 million. The samples had a height of around 170 mm and a diameter of 150 mm. The height-to-diameter ratio of the specimens must be 1.5, achieved by coring the specimen to reduce the diameter from 150 mm to 100 mm and trimming the core ends to reduce the height from 170 mm to 150 mm.

### 2.5. Dynamic Modulus (|E*|) Test

The dynamic modulus test applies a sinusoidal load to the specimen to measure |E*| and phase angle (Ø) at specified temperatures and frequency bands [51]. This test was conducted using UTS 6 Software installed in AMPT. The AMPT used for this research is manufactured by IPC Global. Studs or gauge points are attached to the sample with the help of a gauge point fixing jig that is designed to maintain the required gauge length of 70 mm for the studs. Axial strains and deformation in the specimen are measured with the help of LVDTs (linear variable differential transformers) fixed on these studs. The specimens are placed in an environmental chamber before testing for temperature equilibrium.

In this study, six frequencies (25, 10, 5, 1, 0.5 and 0.1 Hz) were selected, and the specimens were conditioned and loaded at four temperatures (4.4, 21.1, 37.8, and 54.4 °C). Three samples were prepared and tested for each CRM SMA percentage. |E*| and Ø were reported by the software with respect to the frequencies selected at each test temperature.

### 2.6. Dynamic Modulus Master Curves

Master curves are developed from the dynamic modulus results obtained from UTS 6 software, as recommended by AASHTO TP 62 [51]. Master curves are produced in the Microsoft Excel Spreadsheet, master solver, developed by Ramon Bonaquist, that fully characterizes asphalt mixtures. This is accomplished through the time–temperature superposition principle that employs a shift factor to transfer the |E*| values obtained at different temperatures to the reference temperature (21.1 °C). The adjustment needed to bring the data value to the reference temperature is explained by the shift factor.

### 2.7. Performance Modeling

#### 2.7.1. Non-Linear Regression

The performance prediction model was generated in SPSS version 23 using |E*| as the performance indicator. The functional form selected for the model was Cobb–Douglas, which is widely used in cost-based empirical studies and to identify interactions among two or more variables with different functional forms [52]. The Cobb–Douglas model, in its general, generic functional form, is shown in Equation (1).

(1) $$Y=\alpha \;x\;X_{i}^{\beta_{i}}$$

i represents the number of variables, i.e., 1, 2, 3, …, n

#### 2.7.2. Artificial Neural Networks (ANNs)

The foundation of deep learning, a branch of machine learning, is formed by neural networks. The organization of the human brain influences the programming of neural networks [34,53]. Data are input into neural networks, which learn the structure of the data during training and produce outputs for new input values based on that training. Neural networks consist of layers composed of neurons, the core processing units. There are three types of layers: input, output, and hidden layers. Connections between neurons in different layers are mediated by channels. The input is received by the input layer, which transfers the data to the hidden layers for training and the computations required to predict the output, which is then fed to the output layer. Input and output variables are analyzed, and relationships are determined using computational algorithms, known as activation functions, found in the hidden layers. ANNs can identify the exact framework and relationship between input and output data.

## 3. Results and Analysis

### 3.1. Dynamic Modulus Results

**Figure 3:**
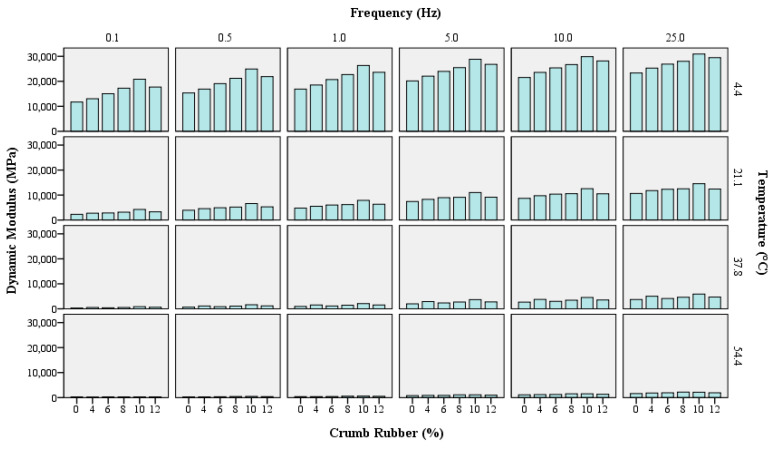
Dynamic modulus results.

The effect of adding CR on the rutting and fatigue performance and viscoelastic behavior of HMA and SMA mixtures has been evaluated extensively in the literature, resulting in enhanced pavement performance in terms of these parameters [20,22,54,55]. The effect of CR on the dynamic modulus of traditional HMA had also been evaluated in past studies, resulting in improved dynamic modulus performance [32,33,56,57]; however, the current body of knowledge is devoid of evaluating the effect of CR on the dynamic modulus of SMA mixtures [30]. In this study, three replicates were tested for each CR percentage (0%, 4%, 6%, 8%, 10%, and 12%) added to the mix, at four temperatures and six frequency sweeps. Samples were brought to the test temperature in a conditioning chamber prior to being loaded into the AMPT test chamber. Average |E*| results for the three replicates are shown in Figure 3 with respect to temperature and frequency. It can be seen that the addition of CR has increased |E*|, thereby improving stiffness. This increase in dynamic modulus can be attributed to the higher stiffness added by CR, with the optimum percentage being 10% for SMA 19. Although the dynamic modulus exhibited a peak at 10% CR, statistical analysis (ANOVA with post hoc Tukey tests) indicated no significant differences among 8%, 10%, and 12% CR contents, suggesting that the identified optimum represents a peak trend within the tested range rather than a statistically distinct maximum. The variation in |E*| with the addition of CR in SMA shows the sensitivity of |E*| to variation in CR percentage in SMA. The interaction between loading frequencies and temperatures in CRM SMA mixtures reveals a complex yet beneficial relationship, in which the addition of CR at an optimum quantity (10%) improves dynamic modulus performance.

As an initial step in evaluating the stiffness of asphalt concrete mixtures, researchers evaluated the effect of temperatures and loading frequencies on the dynamic modulus of HMA mixtures [32,34,58]. This study also evaluated the effects of temperature and frequency on the dynamic modulus of CRM SMA mixtures. Results indicate that |E*| is sensitive to higher temperatures, with a significant reduction in stiffness observed above 37.8 °C. This behavior can be attributed to the temperature sensitivity of strain, which increases at higher temperatures, leading to greater flexibility and reduced stiffness. Conversely, lower temperatures reduce strains, resulting in higher stiffness of the mix [22]. At higher frequencies, we see a notable rise in |E*| values due to the stress–strain relationship of the modulus, as lower contact pressure and time of the tire on pavement surfaces result in higher dynamic modulus values.

### 3.2. Isothermal and Isochronal Curves

**Figure 4:**
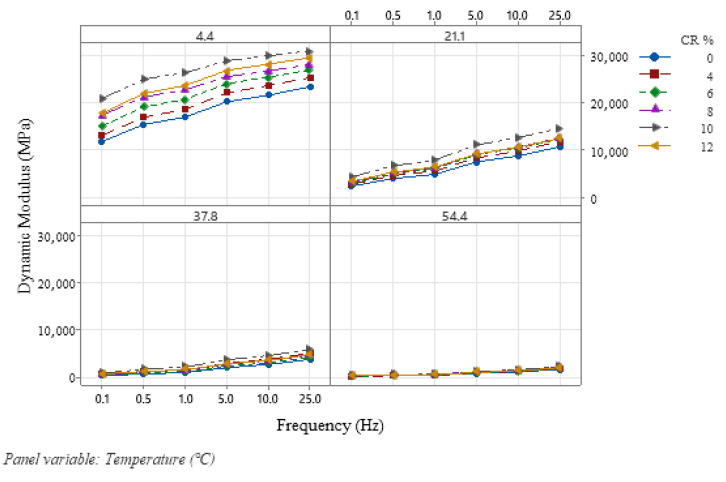
Isothermal curves for dynamic modulus.

Isothermal and isochronal curves graphically present the sensitivity of |E*| to frequency and temperature. Irfan et al. [59] studied the effect of temperature and frequency on the dynamic modulus of Hot Mix Asphalt (HMA) for wearing and base course. Some other notable studies also included this analysis in the dynamic modulus behavior on virgin HMA samples and CRM HMA samples [32,34,54]. As a fundamental step in the dynamic modulus evaluation of asphalt concrete mixtures, this study also prepared isothermal and isochronal curves for CRM SMA samples to understand the effects of temperature and frequency at different CR addition percentages. It is noted from the isothermal curves of |E*| (Figure 4) that, for a constant temperature, a significant increase is recorded in the |E*| values with rising frequencies, which stipulates the sensitivity of |E*| to frequency. At higher frequencies, we see a notable increase in |E*| values due to the stress–strain relationship of the modulus, as lower contact pressure and time of the tire on pavement surfaces results in higher |E*| values. The higher the loading frequency, the higher the |E*|.

**Figure 5:**
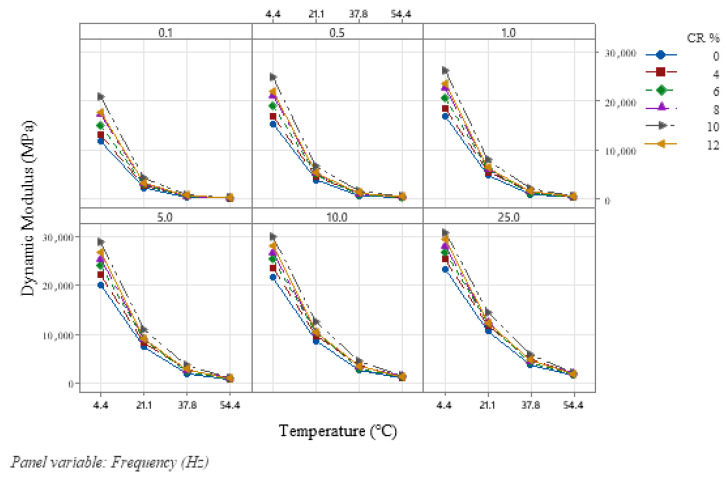
Isochronal curves for dynamic modulus.

It can be inferred from the isochronal curves (Figure 5) that, as the temperature rises, the |E*| decreases while keeping frequency constant. There is a significant decrease in |E*| at higher temperatures, as shown by the curves, indicating that |E*| is highly sensitive to temperature. Higher |E*| values are noted at low temperatures, as can be deduced from the stress–strain relationship, as the strain is decreased at lower temperatures due to higher stiffness. Lower |E*| values are reported at high temperatures, as strain increases with greater flexibility.

### 3.3. Dynamic Modulus Master Curves

**Figure 6:**
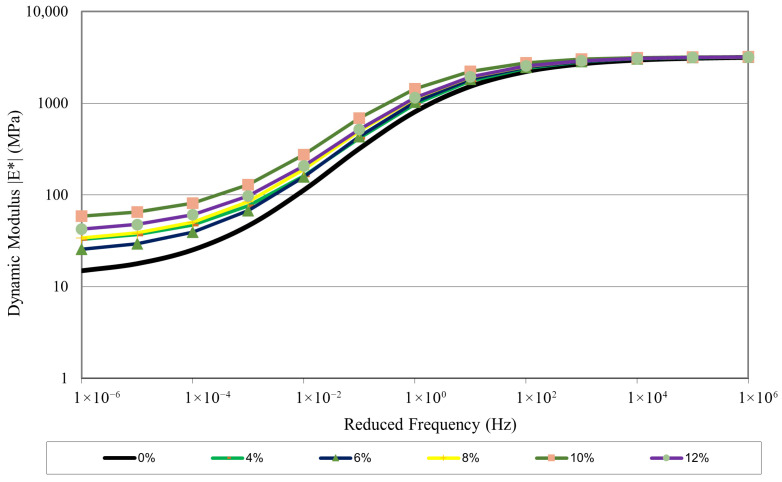
Master curves.

Master curves were produced for each CRM SMA mixture (0%, 4%, 6%, 8%, 10%, and 12%) to characterize pavement behavior under different temperature and loading conditions. Master curves were generated at 21 °C, which was the reference temperature. Figure 6 displays the master curves for all tested samples, indicating that the addition of CR has improved the mixture’s performance. The addition of CR has improved the |E*| values of the mix in high-temperature and low-frequency regions, thus making the mix more rut-resistant. Lower frequencies correspond to lower speeds and longer tire–pavement contact times, whereas higher frequencies correspond to higher speeds and shorter contact times. It can be discerned from the master curves that, as the frequency increases, |E*| also increases. Variation in the mixtures decreases with increasing frequency, as seen in the master curves. It is evident from the master curves that 10% CRM SMA exhibits better performance and greater stiffness across all frequencies, while the neat mixture shows the lowest performance. So, 10% CRM SMA is more rut-resistant as compared to other mixtures, as it is relatively stiffer in the high-temperature region of the master curves.

### 3.4. Statistical Design of Experiment for |E*|

Statistical software (Minitab version 21) was used to identify the variables influencing |E*| values using a two-level Factorial Design. The variables selected for the design, along with their high and low levels, are shown in Table 9. The experiment was designed with a 95% confidence level and a significance level of 0.05.

The effect estimates of temperature, frequency, and CR for |E*| are presented in Table 10, which infers that |E*| is most affected by temperature, whereas the other factors, i.e., frequency and CR, have less effect on the response variable as compared to temperature, though all three variables are significant. The nature of the relationship is inferred by the arithmetic sign of the effect estimate. It is evident from the effect estimates that a rise in temperature from low to high will decrease |E*|, and vice versa, whereas a rise in frequency and CR will increase |E*|, and vice versa. It should be noted that the factorial analysis considered only two temperature levels (4.4 °C and 54.4 °C); therefore, the observed trend reflects the response within the tested range and does not imply a linear relationship between temperature and |E| across the full temperature spectrum. Table 10 also presents the two-way and three-way interaction effects of these three variables. It can be inferred that the two-way interaction effects of temperature–frequency and temperature–CR% were significant, whereas the frequency–CR% interaction was not. The statistically insignificant interaction between frequency and CR% indicates that the stiffening and elastic effects of crumb rubber modification in the SMA (characterized by the dominance of stone-to-stone skeleton) mixture remain relatively consistent across the tested range of loading frequencies. Similarly, the three-way interaction was also insignificant. The statistically insignificant three-way interaction (Temp × Freq × CR%) indicates that no pronounced higher-order effect exists within the tested range, with mixture behavior primarily governed by main and two-way interaction effects rather than by complete independence among variables. The significant interaction effect between temperature and CR% suggests that the dynamic modulus may vary with temperature differently depending on the amount of crumb rubber added.

**Figure 7:**
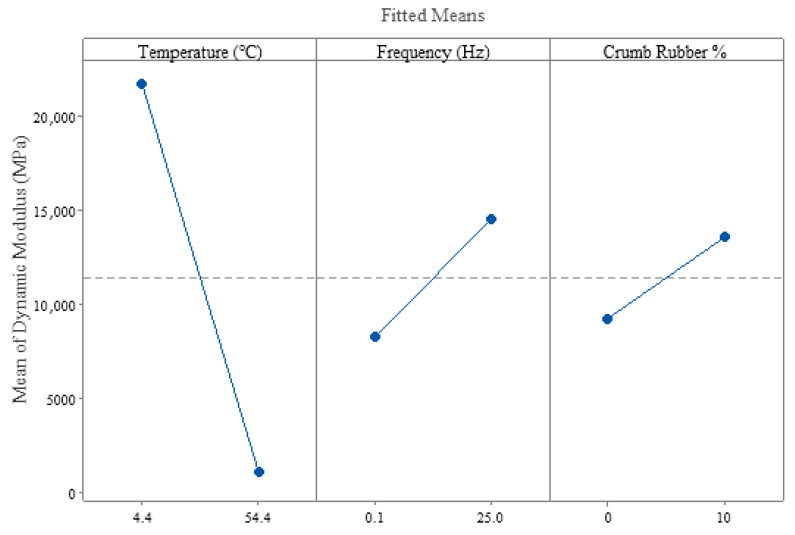
Main effects plot for dynamic modulus.

The plot of the main effects for |E*| is displayed in Figure 7 where the fitted means of |E*| for low and high levels of the factors are plotted to show the strength of the relationship, which can be judged by the slope of the line connecting the low and high levels. Temperature has a sharp slope, which indicates a strong relationship or effect on |E*|, and the descending direction of the slope indicates its inverse relationship. Frequency and CR content show a direct and strong relationship with |E*|, but less as compared to temperature.

**Figure 8:**
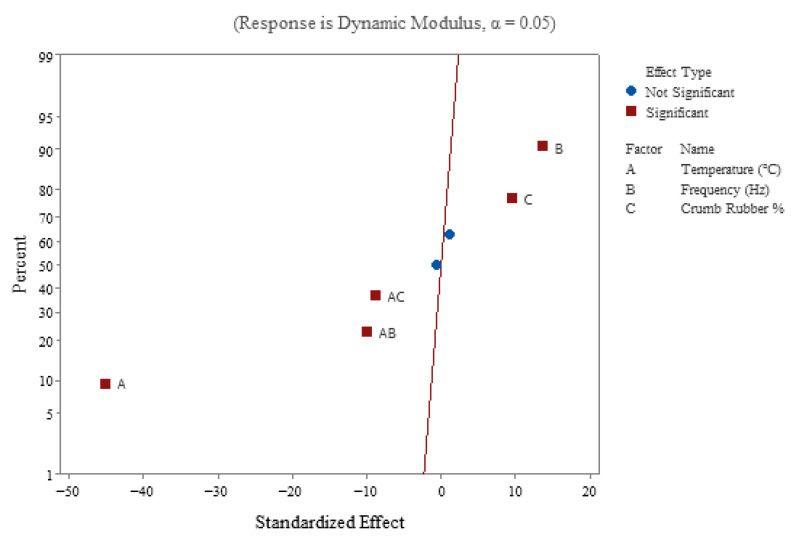
Normal plot of standardized effects for |E*|.

A normal plot of standardized effects (Figure 8) indicates whether a factor is significant (red squares) or otherwise (blue dots). The significant influence of temperature, frequency, and CR as main effects is indicated by red squares. The two blue dots on the normal plot represent the insignificant interaction effect of “frequency and CR” and the three-way interaction of variables. The interaction effects of temperature–frequency and temperature–CR% are significant. The significant interaction effect of temperature–frequency indicates that, at higher temperatures, the SMA samples may behave differently at varying frequencies than at lower temperatures. The significant interaction effect of temperature–CR% means that, at low CR% (say 5%), the |E*| might decrease significantly with an increase in temperature, as compared to the addition of higher CR%. In conclusion, the decrease in |E*| might be less pronounced at higher temperatures due to the addition of CR, as the rubber retains some stiffness. Frequency and crumb rubber lie to the positive side of the reference line, which infers the direct relationship or effect of both these factors on |E*|. The temperature lies to the left of the reference line, indicating an inverse relationship between temperature and |E*|. The distance from the reference line indicates that temperature has a stronger effect on |E*| than the other factors.

**Figure 9:**
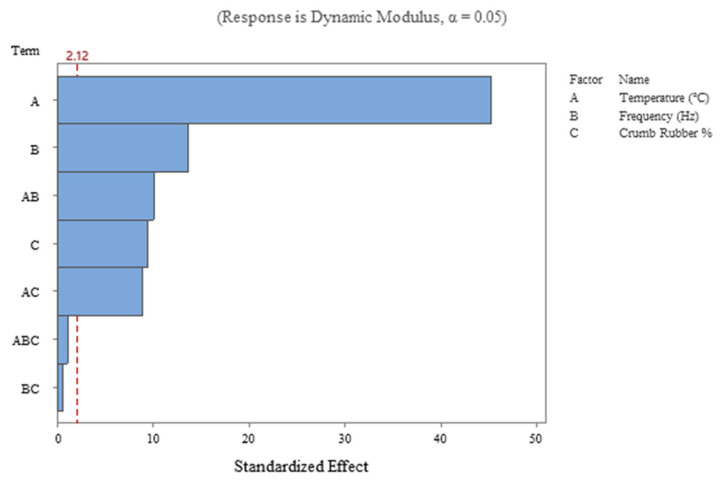
Pareto chart of standardized effects for |E*|.

The comparative significance of the main and interaction effects of factors included in the design are shown in the Pareto chart (Figure 9) in descending order. The standardized effect of each factor on the mean response at a 5% significance level is shown by bars plotted against a reference line indicating the t-critical value. The main effects of temperature, frequency, and crumb rubber are significant, as the bars representing these effects are well above the line, indicating their influence on |E*|. The two-way interactions between temperature and frequency, and between temperature and CR, also cross the line, indicating their effects on dynamic modulus. The bars falling below the reference line include the two-way interaction between frequency and CR, as well as the three-way interaction among all three factors, indicating that these two interaction effects are insignificant.

**Figure 10:**
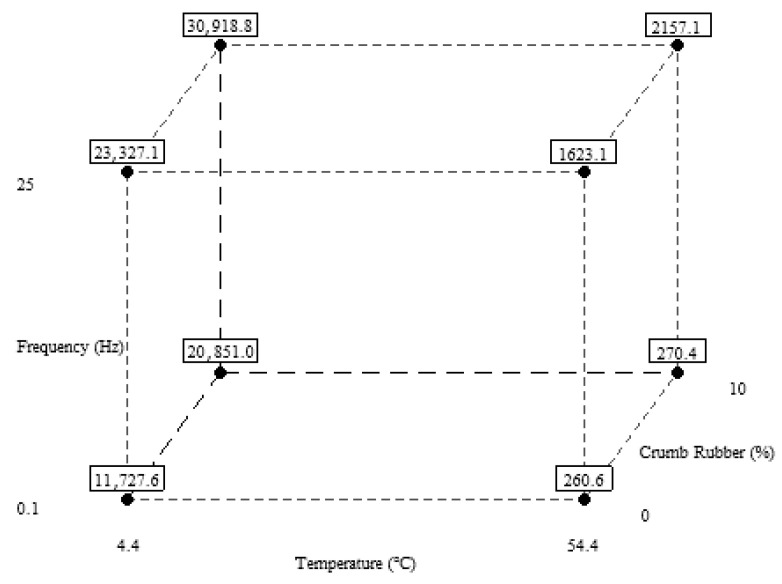
Cube plot for |E*|.

It can be concluded from the cube plot (Figure 10) that a temperature of 4.4 °C, a frequency of 25 Hz, and a CR percentage of 10% produce the highest values for the dynamic modulus. By contrast, a temperature of 54.4 °C, a frequency of 0.1 Hz, and a CR percentage of 0% yield the lowest dynamic modulus values. The reason the dynamic modulus is high at low temperatures is that the increased stiffness reduces the strains induced in the sample. In contrast to low temperatures, the material loses its stiffness and becomes more flexible at high temperatures, producing more strains, resulting in a low dynamic modulus value [22,35]. The cube plot shows that changes in |E*| are more pronounced with temperature than with frequency and CR percentage. The cube plot also confirms that all three factors influence dynamic modulus.

### 3.5. Performance Modeling for Dynamic Modulus

Temperature, frequency, and CR percentage are three parameters that can help predict the dynamic modulus. In this research, the pavement response, expressed with dynamic modulus as the performance indicator, can be predicted using factors that lead to changes in dynamic modulus. For longer pavement life and better prediction of the distress the pavement is exposed to, pavement performance must be modeled at the design stage. Performance modeling was done using an Artificial Intelligence (AI)-based ANN prediction model and a non-linear regression model. Data are generally divided into training, validation, and test datasets. The predicted and experimental results were correlated for both models using the correlation coefficient, which indicates how well a line fits the data. The coefficient of determination was calculated for the models to quantify the proportion of variability in |E*| explained by the independent variables in each model.

#### 3.5.1. Performance Prediction Using Non-Linear Regression

The model was selected using a hit-and-trial method by plotting the dynamic modulus in scatter plots to identify the best relationship between the response variable and its factors. The Cobb–Douglas functional form was selected after evaluating multiple non-linear alternatives (logarithmic, exponential, and power models), as it demonstrated superior predictive stability and reduced overestimation during validation, particularly at higher |E| values. Its multiplicative and log-linear structure enables effective representation of multi-variable interactions while providing interpretable elasticity coefficients, making it well-suited to capturing the viscoelastic behavior of crumb rubber-modified SMA mixtures. For this study, the Cobb–Douglas functional form is transformed as shown in Equation (2) below.

(2) $$\left|E^{*}\right|=\alpha \;x\;T^{\beta_{1}}\;x\;F^{\beta_{2}}\;x\;C^{\beta_{3}}$$

where

*T* = temperature, °C;

*F* = frequency, Hz;

*C* = crumb rubber, %;

*α*, *β*_1_, *β*_2_, *β*_3_ = regression coefficients.

The statistics for the produced model are shown in Table 11.

The produced model has a coefficient of determination (R^2^) of 0.753, indicating that 75.3% of the variance in |E*| is explained by the model, and its predictive capability is satisfactory. The negative sign of the estimate indicates an inverse relationship between the parameter and |E*|. In this case, the parameter estimate for temperature is negative, indicating an inverse relationship between temperature and dynamic modulus. It should be noted that the temperature range considered in this study varies from 4.4 °C to 54.4 °C and therefore does not include zero or negative values. As a result, the power-law formulation with a negative temperature exponent remains mathematically valid within the investigated range. For applications involving temperatures approaching or falling below zero, using an absolute temperature scale (e.g., Kelvin) is deemed more appropriate.

**Figure 11:**
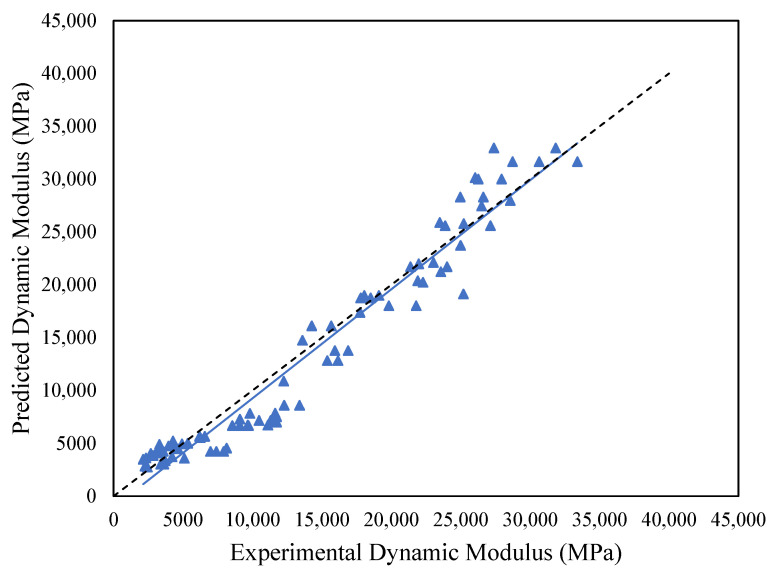
Experimental vs. predicted |E*| for regression model.

Model evaluation was conducted using the Mean Absolute Percentage Error (MAPE). The study utilized 20% of the data to validate the regression model. The MAPE estimated for the model is 18.49%, which is good but not excellent. Figure 11 shows the validation plot of the model with the observed or experimental values on the horizontal axis and predicted ones on the vertical axis to show the predictive capability of the model. The developed model shows good performance, as its values are mostly close to the 45° line shown in dotted lines. The observed deviation at higher |E| values is attributed to the Cobb–Douglas model’s assumption of constant elasticity, which limits its ability to fully capture the highly non-linear viscoelastic response of SMA mixtures under extreme stiffness conditions, i.e., at low temperatures and high frequencies.

#### 3.5.2. Performance Prediction Using Artificial Neural Networks

**Figure 12:**
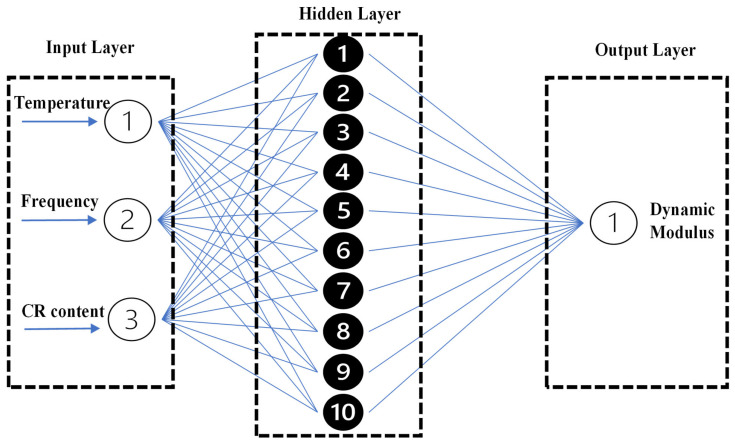
Proposed ANN architecture.

ANN employs training algorithms inspired by the human brain’s framework. Three algorithms are commonly used for performance modeling with ANNs: Levenberg–Marquardt Back Propagation (LMBP), scaled conjugate gradient, and Bayesian regularization. The LMBP algorithm was adopted for training the ANN model due to its robustness and efficiency in solving non-linear optimization problems. Previous studies have shown that the Levenberg–Marquardt optimization approach can significantly improve parameter estimation accuracy and model fitting for viscoelastic material behavior [60]. Data were split into training, validation, and test sets. Weight and gradient computations were performed on the training dataset, while cross-validation was conducted on the validation dataset as training progressed to assess training quality. Cross-validation was performed to control overfitting, which occurs when the algorithm focuses on the details of the training dataset at the expense of losing generalization. Testing or the holdout dataset is used for testing the final trained model, which has been developed using the selected algorithm. The network was trained on 70% of the data, and the remaining 30% was split equally between validation and test sets. A single hidden layer with 10 neurons was used in the network [61]. Although the experimental data include grouped conditions (CR content, temperature, and frequency), random splitting was used to ensure balanced representation across the datasets; however, partitioning by these groups may be considered in future studies to further assess model generalization. The artificial neural network (ANN) architecture is shown in Figure 12, and Table 12 presents the parameter settings for the ANN model.

ANNs are well-known for their ability to approximate any continuous function when provided with appropriate training data. The ANN model efficiently captures complex non-linear relationships between the variables. This makes ANNs suitable for predicting the dynamic modulus behavior of CRM SMA mixtures, which display non-linear and complex patterns. This research used 432 data points to develop the ANN Model. The data points covered under this study account for 144 instances, include six mix types incorporating different percentages of CR in SMA (0%, 4%, 6%, 8%, 10%, and 12%), four temperature ranges (4.4, 21.1, 37.8, and 54.4 °C) and six frequency bands (25, 10, 5, 1, 0.5 and 0.1 Hz). Three samples for each instance were prepared, and the results were incorporated into the training, validation, and testing of the ANN model. To mitigate overfitting and enhance the reliability of the ANN model, the study applied several best practices, including proper data preprocessing, normalization, and cross-validation. Some previous studies have also developed ANN models to predict various performance measures in pavement engineering using comparable numbers of data points [6,52,62]. It is pertinent to mention that this study also developed a non-linear regression model for dynamic modulus to compare the results of both models for CRM SMA.

Regarding the applicability of the ANN model, it can accurately predict the dynamic modulus of CRM SMA mixtures within the provided range of CR contents and SMA 19 gradation. This property of ANNs for local applicability is demonstrated by a comprehensive study by Barugahare et al. [63], wherein the authors evaluated ANN-based dynamic modulus models on South Carolina asphalt mixtures and compared them with traditional regression models. Results showed that ANN models outperformed regression approaches, and that locally developed ANNs provided more accurate predictions of the dynamic modulus than globally calibrated models.

**Figure 13:**
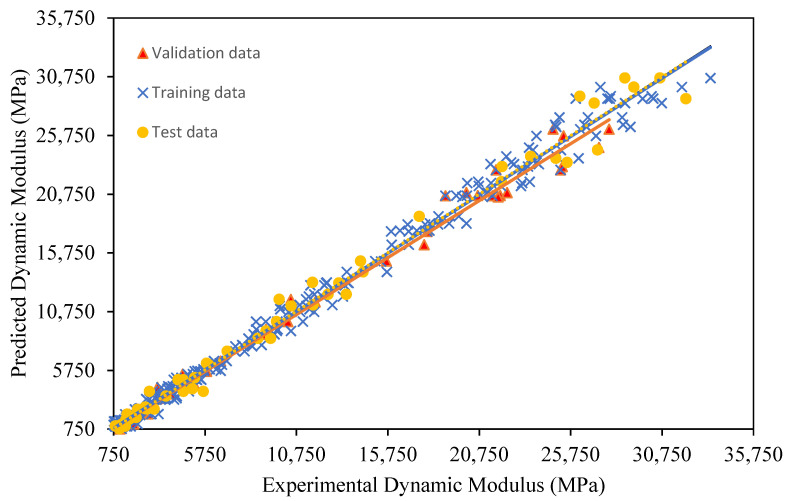
Correlation of experimental and predicted data.

In this study, temperature, frequency, and CR content were used as inputs in the input layer of the ANN model for |E*|. The ANN algorithm was effectively implemented in MATLAB R2024b version, using data with three replicates for each combination of input parameters. Four testing temperatures, six frequency sweeps, and six CR content percentages were used to develop the model and predict dynamic modulus using artificial neural networks. The coefficient of determination (R^2^) for the ANN model is 0.98, indicating that 98% of the variability in |E*| is explained by the model. A comparison of experimental and predicted data is shown in Figure 13. The correlation coefficients for the three datasets indicate that the predicted data are very close to the experimental results. The correlation coefficient (R) for the training, validation, and test datasets is 0.996, 0.996, and 0.994, respectively. These values are very close to 1, which indicates a very strong correlation between the observed and predicted data. Other assessment parameters also show close agreement between the predicted and experimental data, as Mean Absolute Error (MAE) and Root Mean Square Error (RMSE) are low (Table 13).

**Figure 14:**
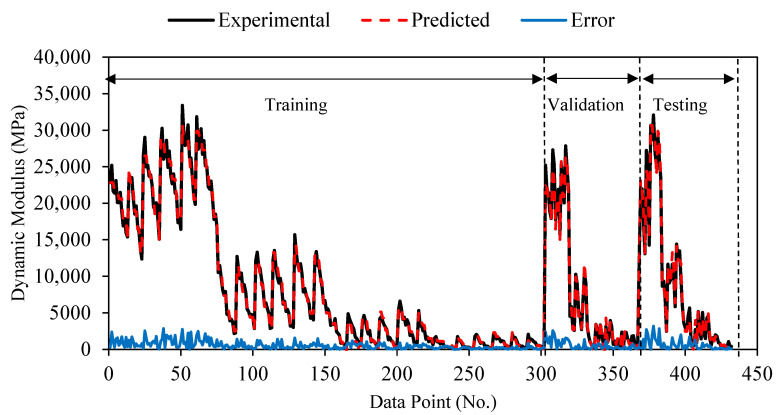
Prediction performance of the |E*| ANN model.

The prediction performance of the ANN predicted model for the dynamic modulus can be seen in Figure 14 as the predicted values lie close to the experimental results. In total, 70% of the data points are included in the training dataset, and the remaining 30% are included in the validation and test datasets. The data are very closely related across all three datasets, as indicated by the prediction performance graphs. The error of the ANN model is plotted in the prediction performance chart alongside the actual and predicted values. The majority of the data points lie close to zero, indicating minimal error and that the model is adequate.

## 4. Conclusions and Recommendations

Conclusions are drawn for the study based on the experimental evaluation and statistical analysis of the results. The dynamic modulus of the SMA mixtures exhibited a peak response at 10% CR modification across all combinations of temperature and frequency sweeps. Higher |E| at intermediate temperatures (21.1 °C) suggests potentially improved rut resistance, though direct rutting tests would be required for validation. The addition of 10% CR to the mixture resulted in a 64% increase in dynamic modulus (on average) compared to the neat mixture, which signifies the stiffness potential of the rut-resistant mix. This 10% CR optimum should be interpreted specifically for the selected materials, mix design, and testing conditions, and higher CR contents may be viable under different mixture configurations and performance requirements. Temperature, frequency, and CR content significantly affect the response variable, i.e., |E*|. An increase in temperature results in a decrease in |E*|, and an increase in frequency dictates an increase in |E*|. The sensitivity analysis of the dynamic modulus shows that a change in temperature from 21.1 °C to 37.8 °C results in a 65% decrease in the dynamic modulus at a given frequency, and a change in frequency from 0.1 to 25 Hz results in a 72% increase in the dynamic modulus at a given temperature. Performance modeling using artificial neural networks (ANNs) yielded a coefficient of determination (R2) of 0.98, compared with 0.75 for non-linear regression. ANNs produced a better model than the non-linear regression model, as it showed a stronger correlation between experimental and predicted data.

Performance tests other than dynamic modulus can be conducted on CRM SMA to further characterize the mixtures. These tests include the flow number, flow time, Hamburg wheel tracker, etc. This study did not incorporate other important variables in the non-linear regression and ANN models developed, e.g., the percentage and type of asphalt, the mixture gradation, and the aggregate type, to obtain a more robust prediction of the dynamic modulus for CRM SMA. These parameters were considered in the initial experimental design to determine the dynamic modulus of CRM SMA mixtures, including a 60/70 penetration binder, an optimum asphalt content of 6.2%, SMA19 gradation selected from NCAT and NCHRP project 9-8, and limestone aggregates from Babozi quarry. However, these parameters were not incorporated in the statistical and ANN model development due to their standard fixed values. Future research can prepare more heterogeneous CRM SMA mixtures by accounting for all these variables, resulting in more robust predictions of dynamic modulus. The study used only a 60/70 penetration-grade binder, which limits its applicability to other binder grades. Future research could utilize 40/50 and 80/100 penetration-grade binders, in addition to 60/70, to broaden applicability. The developed ANN model is applicable only to SMA 19 with a 60/70 binder and 6.2% asphalt content, thereby limiting its practical utility for broader pavement design applications. This study used 432 data points to develop non-linear regression and ANN models. Future research can use more diverse SMA samples and larger datasets to improve the model’s robustness and generalizability. With appropriate retraining of the input–output variables, future studies can use the ANN framework to predict the dynamic modulus of conventional SMA mixtures and with other modifiers, e.g., SBS [64,65], ethylene vinyl acetate (EVA) [66,67], Polyethylene Terephthalate (PET) [68,69], and high-density polyethylene (HDPE) [70]. While crumb rubber modification enhances stiffness and rutting resistance, its potential impact on low-temperature thermal cracking and fatigue damage behavior, particularly in relation to internal skeletal structure and force chain evolution under repeated loading, was not evaluated in this study and warrants further investigation.

## Figures and Tables

**Table 1:** Aggregate properties.

Test		Results	Specification	Standards
Los Angeles Abrasion		22.6	45% (Max)	ASTM C 131 [38]
Flakiness Index		8.79	15% (Max)	ASTM D 4791 [39]
Elongation Index		5.16	15% (Max)	ASTM D 4791 [39]
Aggregate Impact Value		15.8	30% (Max)	BS 812 [40]
Water Absorption	Fine Aggregate	2.37	3% (Max)	ASTM C 128 [41]
	Coarse Aggregate	0.27	3% (Max)	ASTM C 127 [42]
Specific Gravity	Fine Aggregate	2.711	-	ASTM C 128 [41]
	Coarse Aggregate	2.704	-	ASTM C 127 [42]

**Table 2:** Binder test results.

Test	CR Percentage						Standards
	0	4	6	8	10	12	
Penetration Test @ 25 °C	63	45	43	39	36	30	ASTM D5 [43]
Flash Point (°C)	261	271	267	263	258	267	ASTM D92 [44]
Fire Point (°C)	279	283	276	271	269	272	ASTM D92 [44]
Softening Point (°C)	50	52	53	55	57	60	ASTM D36 [45]

**Table 3:** Properties of CR.

Property	Measured Value	Standards
Specific Gravity	1.23	1.1–1.3
Moisture (%)	0.0	<1
Metal (%)	0.008	<0.05
Fiber (%)	0.075	<1
Carbon Black (%)	41	≥28

**Table 4:** Material characteristics of VIATOP^®^ premium.

Properties	Results	Specification
ARBOCEL ZZ 8-1 (%)	88.3%	~90%
Bitumen (%)	11.7%	~10%
Pellet length (Avg.) (mm)	7 mm	2–8 mm
Pellet thickness (Avg.) (mm)	3 mm	3–5 mm
Bulk density (in accordance with DIN EN ISO 60)	472 g/L	440–510 g/L
Finer than 3.55 mm (Sieve analysis)	2.2%	Max. 10%

**Table 5:** Density and VCADRC for trial blends.

Blend	γ_S_ (Kg/m^3^)	γ_W_ (Kg/m^3^)	VCA _DRC_ (%)
Trial Blend I	1606	998	40.5
Trial Blend II	1622	998	39.9
Trial Blend III	1629	998	39.6

γ_S_ represents the unit weight (dry rodded condition) for the coarse aggregate fraction.

**Table 6:** Volumetrics of three trial gradations.

Property	Trial Blend I	Trial Blend II	Trial Blend III
G_mb_	2.378	2.379	2.383
G_mm_	2.466	2.459	2.455
V_a_ (%)	3.6	3.3	2.9
VMA (%)	18.1	18.0	17.9
VCA _MIX_ (%)	35.9	39.1	40.7
VCA _DRC_ (%)	40.5	39.9	39.6

G_mb_ is bulk-specific gravity, and G_mm_ is the theoretical maximum specific gravity of specimens.

**Table 7:** Volumetrics of trial specimens for optimum asphalt content.

Property	Asphalt Content		
	5.5	6	6.5
G_mb_	2.357	2.369	2.378
G_mm_	2.495	2.479	2.466
V_a_	5.5	4.4	3.6
VMA	17.9	17.9	18.1
VCA	35.7	35.8	35.9
Stability	9.76	11.29	8.32

**Table 8:** Mix design for SMA 19.

Mixture Property	Design	Requirement [36]
G_sb_	2.714	-
Asphalt Content (%)	6.2	6.1 min
Stabilizers (%)	0.30	0.30
V_a_ (%)	4.0	4.0
VMA (%)	18.0	17.0 min
VCA mix (%)	35.8	40.5 max
Stability (kN)	10.4	6.2 min

**Table 9:** Factors selected for design.

Factors	Abbreviations	Units	Low Level	High Level
Temperature	A	°C	4.4	54.4
Frequency	B	Hz	0.1	25
Crumb Rubber	C	%	0	10

**Table 10:** Effect estimates of temperature, frequency, and CR for |E*|.

	Main Effects	Effects	*p*-Value	Two-Way Interaction	Effects	*p*-Value	Three-Way Interaction	Effects	*p*-Value
|E*|	Temp	−20,628	<0.001	Temp×Freq	−4605	<0.001	Temp×Freq×CR%	514	0.276
	Freq	6229	<0.001	Temp×CR%	−4043	<0.001			
	CR %	4315	<0.001	Freq×CR%	−252	0.588			

**Table 11:** Summary of the Cobb–Douglas non-linear regression model.

Parameter	Estimate	Std. Error	t-Stat	R^2^ (%)	95% Confidence Interval	
					Lower Bound	Upper Bound
A	49,035.307	6034.823	8.125	75.3	37,173.728	60,896.886
β_1_	−0.857	0.035	−24.48		−0.925	−0.789
β_2_	0.102	0.011	9.27		0.081	0.124
β_3_	0.219	0.051	4.29		0.118	0.320

**Table 12:** ANN model parameters.

Parameter	Setting
Training data (%)	70
Validation data (%)	15
Testing data (%)	15
Hidden layers (No.)	1
Neurons in hidden layer (No.)	10
Training Algorithm	Levenberg–Marquardt
Sampling/Data division	Random

**Table 13:** Evaluation metrics for the developed ANN model.

Training Data	Validation Data	Test Data
R = 0.996	R = 0.996	R = 0.994
MAE = 525.8	MAE = 547.85	MAE = 635.98
RMSE = 759.43	RMSE = 820.21	RMSE = 965.34

## Data Availability

The data presented in this study are available on request from the corresponding author.
